# A comparison of medical and pharmacy student perspectives of a clinical interprofessional home-visit versus a virtual interprofessional workshop

**DOI:** 10.12688/mep.19510.1

**Published:** 2023-05-12

**Authors:** Anita B. Major, Yuanyuan Zhou, Catherine L. Hatfield, Kristina M. Little, Natalie M. Mondragon, Anne C. Gill

**Affiliations:** 1 Department of Medicine-Geriatrics, Baylor College of Medicine, Houston, Texas, 77030, USA; 2 Evaluation, Assessment & Educational Research, Baylor College of Medicine, Houston, Texas, 77030, USA; 3 College of Pharmacy, University of Houston, Houston, Texas, 77204, USA; 4 Pediatrics, Baylor College of Medicine, Houston, Texas, 77040, USA

**Keywords:** Interprofessional Education, Learning outcomes, Instructional design, Communication skills

## Abstract

**Background:** No Place Like Home is a clinical interprofessional education (IPE) activity whereby pharmacy and medical students conduct home visits under the guidance and supervision of a clinical preceptor to homebound patients.

**Purpose:** We examined pharmacy and medical student perceptions of mastery of interprofessional competencies during an in-person clinical home visit pre-COVID-19 pandemic versus a virtual IPE learning activity consisting of didactic and case discussions in response to the global COVID-19 pandemic.

**Methods:** We administered the same modified Interprofessional Collaborative Competency Attainment Survey (ICCAS) instrument, which uses a five-point Likert scale, to both the in-person and the virtual IPE students following their learning activity.

**Results:** We received a total of 459 completed survey responses with an overall response rate of 84%. For both groups of students, the in-person format was preferred, however, to our surprise, the results indicated that students in the virtual group reported greater perceived gain in interprofessional skills than students in the in-person group. In addition, pharmacy students perceived greater gain from the interprofessional activity and offered more thoughtful reflections about their experience.

**Conclusions:** Even though both groups of students preferred the in-person visit, the IPE objectives were equally (for medical students) or better (for pharmacy students) absorbed in the virtual environment than the in-person clinical home visit.

## Introduction

Health care educators are tasked with creating and delivering discipline-specific curriculum that prepares learners for their field of practice and fulfilling the learning requirements of their institutions. Like most health care fields, pharmacy and medicine have interprofessional education requirements ( Association of American Medical Colleges, 2022; American Association of Colleges of Pharmacy, 2022) that must meet the standards of their accreditors. As such, we created the No Place Like Home experience with the intention of meeting Interprofessional Education Collaborative (IPEC) ( Collaborative IE, 2016) competencies. While the original in-person clinical home-visit experience was well-received, COVID-19 required a curricular pivot to a virtual workshop platform.

### Background

The No Place Like Home (NPLH) activity is an IPE experience whereby medical students, pharmacy students, and either a physician or a nurse practitioner preceptor provide care as a team to patients in their homes ( Hatfield *et al.*, 2021). This educational activity was first introduced in 2012 at the Baylor College of Medicine (BCM) and the University of Houston College of Pharmacy (UHCOP) and has consistently been one of the most popular and highly rated IPE activities in our curriculum. This activity has been described in detail elsewhere in the literature ( Misra *et al.*, 2016). But briefly, NPLH pairs a medical student with a pharmacy student to spend one eight-hour day making house calls with either a physician or nurse practitioner facilitator ( Misra *et al.*, 2016). Early in this experience, we observed very high ratings of “overall satisfaction” associated with the activity. However, through an in-depth analysis of the evaluations, we discovered that although the students were awed by the clinical aspects of the session, they were mostly indifferent to the IPE objectives. Simply providing an opportunity for medical and pharmacy students to interact with each other was insufficient to meet IPE objectives. Thus, we redesigned the activity and reinforced training of the preceptors to focus explicitly on the IPE intentions. These corrections were successful, and the activity has continually met the IPE objectives ever since. The research described in this paper was performed entirely after these curricular modifications were made.

Like all educational activities, some changes have ensued over the years; however, the impact of the COVID-19 pandemic required the creation of a virtual, rather than in-person, curriculum, mirroring the efforts of many graduate medical institutions around the globe. ( Sleiwah *et al.*, 2020). The need to implement a virtual NPLH IPE activity was both unanticipated and urgent. BCM clinical educators and IPE experts met to brainstorm and develop a virtual activity that would meet the IPE and clinical knowledge objectives in a safe environment. Though research has shown high levels of student engagement in virtual learning platforms, ( Kay & Pasarica, 2019) most notably for their flexibility, ( Dost *et al.*, 2020; Kay & Pasarica, 2019) it has also been reported that students worry that remote learning could negatively impact their ability to develop clinical competencies ( Huddart *et al.*, 2020). A meta-analysis of blended learning involving both face-to-face learning and e-learning demonstrated consistently better effects on knowledge outcomes when compared with traditional learning in health education ( Vallee *et al.*, 2020). Specific to team-based IPE virtual experiences, student attitudes were unchanged when comparing in-person learning activities to a virtual platform ( DelNero & Vyas, 2021). One study by Robertson *et al.* (2021) suggests that IPE collaborative competencies can be met with a virtual platform.

The Interprofessional Education Collaborative (IPEC) is the recognized national authority on interprofessional curriculum. As such, they describe four core domains for IPE curricula: roles and responsibilities, interprofessional communication, teamwork, and ethics and values ( Collaborative IE, 2016). We wanted and needed to know if the student learning objectives of the in-person, clinical IPE activity could be met in a virtual environment. Evaluation of delivery formats with the same learning objectives has important implications for medical educators across the county, especially where schools have multiple sites or campuses, as each site may be impacted by different resources. Specifically, the Liaison Committee on Medical Education (LCME) accreditation standard 8.7 states, “A medical school ensures that the medical curriculum includes comparable educational experiences and equivalent methods of assessment across all locations within a given course and clerkship to ensure that all medical students achieve the same medical education program objectives.” ( LCME, 2021). Therefore, the purpose of this paper is to compare evaluation data from both in-person and virtual IPE experiences to determine if the learning objectives specific to the IPE competencies above can be effectively met in the virtual format.

## Methods

This was a non-experimental, cross-institute cohort study. The data was collected using course evaluation surveys that included Likert-scaled items for quantitative analysis and open-ended questions that allow for a qualitative analysis of text comments. Mixed methods (triangulation model) ( Schifferdecker & Reed, 2009) were used for data analysis. We obtained approval for this study from the Baylor College of Medicine (protocol number: H-50687) and University of Houston (protocol number: STUDY00003318) Institutional Review Boards.

### The in-person curriculum

One pharmacy and one medical student are assigned to perform approximately four house calls with a trained preceptor for an entire day. The preceptor is either a physician specializing in geriatrics or a geriatric nurse practitioner. As a team, they visit patients in their homes, addressing medical, functional, and psychosocial aspects of care and offering patient/caregiver education. Opportunities for students to teach each other occur throughout the day and debriefing in the car between visits has proven a valuable part of the experience ( Hatfield *et al.*, 2021). Preceptors are directed to ask the medical and pharmacy students questions related to the IPE objectives to generate dialogue and reflection.

### Participants

A total of 545 students participated in this project: 276 from Pharmacy (in-person: 125; virtual: 151); and 269 from Medicine (in-person: 180; virtual: 89). They were convenient samples of second- or third-year medicine or third-year pharmacy students who participated in the NPLH program from February 2019 to September 2021 (in-person: February 2019 to January 2020; virtual: October 2020 to September 2021). Other demographic information, such as gender and race is summarized in Table 1.

**Table 1.** Cohort Sample Characteristics.

Pharmacy at UHCOP (n = 201)		Medicine at BCM (n = 258)	
Characteristic	Count (%)	Characteristic	Count (%)
*Expected Graduation Year*		*Expected Graduation Year*	
2020	17%	2020	2%
2021	18%	2021	56%
2022	41%	2022	23%
2023	24%	2023	19%
*Gender*		*Gender*	
Male	33%	Male	42%
Female	67%	Female	58%
*Race*		*Race*	
White	21%	White	44%
Asian	53%	Asian	43%
Hispanic	11%	Hispanic	4%
Black or African American	9%	Black or African American	3%
Other	1%	Other	2%
Unknown	5%	Unknown	3%

### The virtual curriculum

The house call clinicians created a set of real-life cases, each of which illustrates an aspect of care such as deprescribing, recent hospital discharge, chronic care management, new hospice referral, to name a few. Two groups of two-to-five students each, half pharmacy and half medical, were assigned to a zoom call with one preceptor for a two-hour experience. Fewer resources were required to deliver this curriculum, in terms of time (eight versus two hours), resources (medical equipment and transportation), and number of preceptors (4 versus 2). Three cases were reviewed with the preceptor leading the discussion; every group discussed the transition of care case after hospital discharge, and the other cases were determined by the preceptor to offer some variety among the groups. Finally, to increase active engagement of the students, they were given a case to work on together, with the preceptor muted but observing. Debriefing of how they functioned as a team was deliberate in the final activity of the session.

### Evaluation surveys

The evaluation surveys were designed to obtain data on students' perception of their growth in interprofessional skills and the overall learning experience of the NPLH program. For in-person and virtual curriculum, we used the same ten pairs ( *i.e.*, before and after participating in the learning activities) of five-point Likert scale items (1: poor; 5: excellent) that focused on IPE objectives and teamwork in a single survey collected after the experience. We included several open-ended questions focusing on strengths, weaknesses, and learning outcomes of the NPLH experience to elicit a deeper understanding of students' experiences. All medical and pharmacy students were asked to complete the survey after the experience, whether it was in-person or virtual.

### Data analysis

***Quantitative analysis*.** Descriptive statistics were tabulated by delivery format (in-person versus virtual) and discipline (medicine versus pharmacy). Non-parametric comparison (Wilcoxon signed-rank test) and parametric comparison (paired t-test) for related samples were conducted for each paired item to examine statistically significant differences in students' perceptions of interprofessional skills before and after this learning experience. Further statistical analyses were conducted using independent sample t-tests to examine if discipline (medicine versus pharmacy) or delivery format (in-person versus virtual) had an impact on interprofessional growth. Finally, we created two composite variables: Average-Before and Average-After. These two variables averaged the ten pairs of items for Before and After items. ANCOVA were used to examine all factors in an integrated model. We reported the p-values along with the effect sizes (Cohen’s d) for all major statistical analyses. We used SPSS version 28 (IBM Corp, Armonk, New York) to conduct these quantitative analyses.

***Qualitative analysis*.** Qualitative analyses focused on three domains: 1) types of learning that occurred in this experience; 2) superiority of in-person or virtual format; and 3) discipline differences. Two independent reviewers analyzed the text data and reached a consensus on themes for each of the three domains. The text comments were coded based on types of learning, separated by delivery format and discipline. The percentage of themes brought up by students can be compared between delivery formats or disciplines to examine superiority of teaching formats or discipline differences.

## Results

### Quality of data

Pharmacy students, in general, provided higher quality ( *i.e.*, more thoughtful and descriptive) text responses to the series of learning experience questions (85%–100% provided text comments). Medicine students provided fewer responses (49%–99%) to text comment questions, but the available responses were sufficient for investigating our research question.

### Quantitative findings

We received 459 completed survey responses: 201 from Pharmacy (in-person: 71; virtual: 130); 258 from Medicine (in-person: 176; virtual: 82), with the response rates of 57%, 86%, 98%, and 92%, respectively. The mean and SD for pre- and post-experience of each survey item are provided in Table 2, separated by disciplines and delivery formats.

**Table 2.** Descriptive statistics and mean comparison between In-person and Virtual.

Learning Objective Questions	Delivery Format	Before Participating in NPLH						After Participating in NPLH					
		Pharmacy (n=71)		p	Medicine (n=176)		p	Pharmacy (n=130)		p	Medicine (n=82)		p
1.Provide constructive feedback to IP team members	In-person	3.37	0.8	ns	3.60	0.9	ns	3.83	0.8	**	4.03	0.8	ns
	Virtual	3.45	0.9		3.46	0.8		4.20	0.8		4.04	0.8	
2.Seek out IP team members to address issues	In-person	3.56	0.8	ns	3.69	0.9	**	4.03	0.7	*	4.19	0.8	ns
	Virtual	3.47	0.8		3.39	0.8		4.28	0.8		4.00	0.7	
3.Work effectively with IP team members to enhance care	In-person	3.63	0.9	ns	3.80	0.8	ns	4.20	0.7	ns	4.30	0.7	ns
	Virtual	3.52	0.8		3.60	0.8		4.31	0.7		4.13	0.7	
4.Learn with, from and about IP team members to enhance care	In-person	3.65	0.8	ns	3.83	0.8	ns	4.30	0.6	ns	4.36	0.7	*
	Virtual	3.52	0.8		3.66	0.7		4.41	0.7		4.17	0.7	
5.Identify and describe my abilities and contributions to the IP team	In-person	3.61	0.8	ns	3.63	0.8	ns	4.10	0.8	ns	4.16	0.8	ns
	Virtual	3.45	0.9		3.59	0.8		4.28	0.7		4.11	0.7	
6.Understand the abilities and contributions of IP team members	In-person	3.56	0.8	ns	3.64	0.9	ns	4.23	0.7	*	4.32	0.7	ns
	Virtual	3.58	0.8		3.54	0.8		4.42	0.7		4.13	0.8	
7.Recognize how others’ skills and knowledge complement and overlap with my own	In-person	3.62	0.7	ns	3.73	0.8	ns	4.27	0.6	ns	4.34	0.7	ns
	Virtual	3.57	0.9		3.61	0.8		4.43	0.7		4.18	0.7	
8.Use an IP team approach with the patient to assess the health situation	In-person	3.45	0.8	ns	3.68	0.8	ns	4.11	0.8	*	4.23	0.7	ns
	Virtual	3.48	0.8		3.59	0.9		4.37	0.7		4.15	0.7	
9.Address team conflict in a respectful manner	In-person	3.61	0.8	ns	3.94	0.8	ns	4.14	0.8	ns	4.24	0.8	ns
	Virtual	3.68	0.9		3.78	0.9		4.36	0.8		4.18	0.8	
10. Negotiate responsibilities within overlapping scopes of practice	In-person	3.52	0.8	ns	3.60	1.0	ns	4.11	0.8	ns	4.07	0.8	ns
	Virtual	3.43	0.9		3.52	0.8		4.23	0.8		3.98	0.8	

Note. ** indicates group comparison is statistically significant at the 0.01 level; * indicates group comparison is statistically significant at the 0.05 level, “ns” indicates group comparison is not statistically significant at the 0.05 level.

### Effectiveness of the NPLH program

For each of the ten pairs of Likert scale questions, regardless of discipline and delivery format, the post perception mean scores of the post-experience were higher than the mean pre-experience scores. We found statistically significant differences in self-perception of interprofessional skills before and after this learning experience (Wilcoxon signed-rank tests: ps < 0.001 for all ten pairs of questions; Paired t-test: ps < 0.001 for all ten pairs of questions). The results provide evidence that the NPLH program enhances students’ interprofessional skills, regardless of discipline and/or delivery format.

### Difference in disciplines

In general, pre-experience mean scores for pharmacy students were lower than pre-experience mean scores for medical students and, the mean post-experience scores of for pharmacy students were higher than the mean scores for medical students for both in-person and virtual formats.

**Figure 1.**
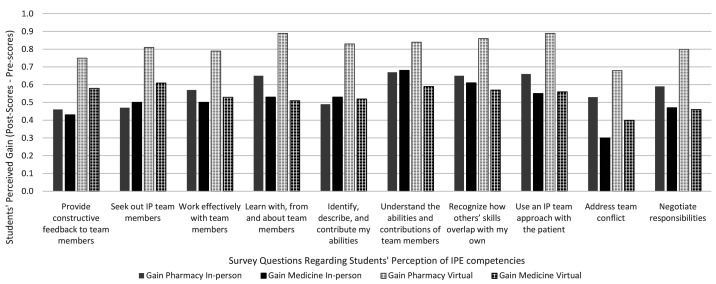
Students’ Perceived Gain in IPE Competencies by Discipline and Pedagogy.

Pharmacy students’ scores demonstrate more perceived gain than those of medical students ( *i.e.*, the difference between students’ post- and pre-experience) in the ten pairs of Likert-scale questions. A series of independent sample t-tests were conducted to examine discipline differences on perceived gain. For the in-person format, one IPE survey item ( *i.e.*, address team conflict in a respectful manner) showed that pharmacy and medical students were statistically significantly different in gaining in IPE competencies (ps: 0.002-0.888, Cohen’s ds: -0.060-0.436). For the virtual format, IPE improvement for pharmacy and medical students was statistically significantly different for all ten survey items (ps: <0.001-0.032; Cohen’s ds: 0.304-0.576) (see Figure 1 for detailed results).

### In-person format versus virtual format

In general, the pre-experience mean scores for the virtual group were lower than those for the in-person group across all ten questions. However, the mean post-experience scores of the virtual group were higher than those of the in-person group. The results might indicate that students in the virtual group reported more perceived gain in IPE skills than students in the in-person group.

More independent sample t-tests were conducted, examining the difference of perceived gain between delivery formats narrowed down by disciplines. The perceived gain in IPE competencies for the in-person and virtual format for pharmacy students was statistically significantly different in nine out of ten survey items (ps: < 0.001-0.127; Cohen’s ds: 0.226-0.563) with the virtual method showing superiority. In contrast, the gain in IPE skills among medical students for the in-person and virtual formats was relatively equivalent with no statistically significant differences. The improvement in IPE proficiencies according to discipline and delivery format is shown in Figure 1.

### Integrated model

Segregate analyses have shown a consistent pattern regarding the effect of discipline and delivery format. To estimate the impact of these two factors, we generated two composite scores: Average-Before and Average-After. We conducted an ANCOVA using the Average-Before as the covariate, discipline, and delivery format as two independent factors. The results indicate that the Average-Before was a statistically significant covariate (p < 0.001, partial Eta squared = 0.554), discipline and delivery format were both statistically significant factors (discipline: p = 0.001, partial Eta squared = 0.022; delivery format: p = .031, partial Eta squared = 0.010).

### Qualitative findings

The findings revealed in the quantitative results are elaborated upon in the qualitative analyses by comparing the difference in coding themes across teaching formats or disciplines.

### Student take aways

We coded the responses to the question, "What learning occurred for you in this experience?" Responses were matched to five themes: Communication, Interprofessional perspectives, Geriatric care, Clinical knowledge, and Others. When we noticed that some themes might need to be divided into even smaller categories, we created lower-level categories/themes to display the difference in learning between in-person and virtual formats. For example, for the theme “communication” we noticed that there might be differences in communicating with professionals or patients, so we created three sub-themes: communication with professionals, communication with patients, and communication in general, to capture the difference across teaching formats. Detailed coding results were showing in Table 3.

**Table 3.** Percentage of themes/sub-themes calculated by the number of quotes divided by the number of text comments to the question, “What learning occurred for you in this experience?”

Themes and example quotes		In-person	Virtual
***Communication***			
Communication with professionals	Example quote: " *I learned to more effectively communicate with other medical professionals.*"		
	Pharmacy	12.70%	10.80%
	Medicine	1.00%	2.40%
Communication with patient	Example quote: " *I learned more about how to talk to patients and their families...*"		
	Pharmacy	14.10%	0.00%
	Medicine	6.80%	0.00%
Communication in general	Example quote: " *I learned how to communicate things concisely and effectively*"		
	Pharmacy	0.00%	3.10%
	Medicine	1.00%	0.00%
***Interprofessional perspectives***			
Roles and responsibilities	Example quote: " *I got to see how some of the med students come up with differential diagnosis...*"		
	Pharmacy	15.50%	24.60%
	Medicine	23.30%	21.40%
Teamwork	Example quote: " *I was able to collaborate with other disciplines and work towards a common * *goal of understanding different aspects of a case study.*"		
	Pharmacy	4.20%	10.80%
	Medicine	3.90%	9.50%
***Clinical Knowledge/skills***			
Clinical Knowledge	Example quote: " *I gained a lot in terms of concrete knowledge (medication side effects, * *polypharmacy)* "		
	Pharmacy	28.20%	27.70%
	Medicine	15.50%	42.90%
Geriatric Care/patient care	Example quote: " *I learned more about geriatric patients and how to care for them.*"		
	Pharmacy	23.90%	20.80%
	Medicine	39.80%	35.70%
***Others***			
Health care system knowledge	Example quote: *"I learned about the barriers to healthcare for many of these home-bound* * patients, including insurance/ financial concerns, lack of mobility and transportation, and poor* * health literacy."*		
	Pharmacy	12.70%	8.50%
	Medicine	17.50%	11.90%
Social economic perspective	Example quote: " *This experience reinforced the importance of understanding the social and* * familial factors in the lives of our patients in order to better tailor treatment to their specific * *situation.*"		
	Pharmacy	11.30%	5.40%
	Medicine	13.60%	0.00%
Preceptors as role model	Example quote: " *I learned [about the impact] professionals in this line of work have on* * patients…how being motivating, caring, supportive, and showing dedication towards our * *patients can [be] very meaningful and positive.*"		
	Pharmacy	7.00%	0.80%
	Medicine	4.90%	2.40%

### Which format was preferred, in-person or virtual?

For the question "Please comment on the areas for improvement for this learning experience," no student who had an actual home visit proposed to switch this program to a virtual format. However, for those who received the training virtually, 24.1% of students at BCM and 13.7% of students at UHCOP indicated that they preferred to have an actual home visit. The results indicated that for both disciplines, if students were given a choice, students would prefer the in-person format to the virtual format.

*“If possible, it would've been helpful to have the experience in person”* (from a medical student)

*“I would have loved to do this in-person instead of virtual.”* (From a pharmacy student)

We observed four common differences across disciplines pertaining to delivery format (in-person versus virtual), and the differences for the pharmacy students were more apparent than the medical students.

1) Communication with patients was not applicable in the virtual format because there was no real patient interaction
2) Students have enhanced interprofessional perspectives in the virtual format
3) Specific clinical knowledge pearls were delivered more effectively in the virtual, case-based format
4) Because actual visits provided students with the real-world experience of observing patients’ challenges and conditions, students gained a more social-economic perspective and health care system knowledge in the in-person format

We observed similar patterns to the questions, "What happened during your experience? What did you observe?" For the in-person format, most students highlighted the home visit. Some students mentioned their observed learning in the home setting related to the medication review and physical exams. For the virtual format, many students mentioned interprofessional collaboration between disciplines. Students also highlighted the case studies and explained what they learned from these cases.

## Discussion

We created a virtual interprofessional educational activity for medical and pharmacy students related to home visits of geriatric patients. The goal of this activitiy was to meet the same IPE learning objectives previously approved by our Curriculum Committees during an international pandemic that protected the safety of the patients, learners and faculty. With the virtual curriculum, we continued to collect data which enabled us to compare the differences in perceived learning as reported by the students. To our surprise, the virtual format was rated equivalent if not more effective in meeting IPE objectives. In addtion, the pharmacy students had greater perceived gains in learning regardless of format.

### Why were IPE learning objectives more effectively met in the virtual format?

Across the entire curriculum, our experience at BCM is that almost all students prefer in-person learning compared to a virtual format. Therefore, we were somewhat surprised to learn that the IPE competencies were more consistently met in the virtual format. This phenomenon may be explained by the fact that the opportunities for non-IPE learning in the in-person home visit offer additional value to the experience but may detract from IPE goals. For example, immersion in the patient’s home enviornment may be the first time the students experience the the socioeconomic realities of underservered populations, and this can overshadow the mechanics of the interprofessional team and team members’ roles.

Likewise in the virtual experience, the faculty were able to create cases that purposely offered opportunities for interprofessional collaboration whereby students of both disciplines could suggest interventions, which, due to the exigencies of the in-person visit, cannot always be assured. Also, in the virtual format, students are given time to work as a group with minimal or no input from the preceptor. Preceptor involvement during the in-person format tends to be more consistent with fewer opportunities to independently work together. The role modeling of interprofessional teamwork is not always possible in the in-person format as we gather in the patients’ homes though it is the culmination of the virtual session.

### Why do pharmacy students seem to benefit more from the NPLH program?

It appears the pharmacy students responded more thoughtfully to the survey in their text comments and provided more insightful thoughts/opinions. They seem to be more appreciative of the learning opportunities to observe how future doctors react to the same cases. This might be due to pharmacy students being third years and earlier in their training than the medical students who were third and fourth years. In addition, medical students have many more clinical opportunities and patient care experiences with different ages and diagnoses. Therefore, their enthusiasm for the NPLH activity may be blunted, and their perception of change may not be as pronounced as that of pharmacy students, as suggested in the comments below: “… *having discussed with non-medical school students, the experience was novel for them. However, for us MS3s [third-year medical students], it was superfluous…after many similar activities during preclinical and clinical studies.”* (from a medical student)

*“I think this overlapped strongly with our ARTS class [second-year medical student basic science course on aging], so it felt like more of a refresher than presenting new information.”* (from a medical student)

### Should we replace home visits with a virtual experience?

As we have observed in previous research ( Hatfield *et al.*, 2021), in-person interprofessional home visits offer unexpected learning experiences that can overpower student emotions and undermine the IPE intentions. While these experiences have learning value in their own right, we must stay vigilant of the IPE objectives of the curriculum and continue to enforce those objectives through faculty-guided reflection and attention to student perceptions. We are confident that both aims can be accomplished as we have demonstrated in our own experience. 

Limitations of this study include the generalizability of our evaluation and research. Our location in a large, urban enviroment and our student demographics may not be reprensentative of a majority of medical and/or pharmacy schools. In addition, not all programs have a county hospital system with a home visit option.

Our results suggest that a virtual session requiring fewer resources and time is just as effective, if not more so, than the in-person home visit in keeping the IPE goals in focus. Yet, as of February 2022, we resumed the in-person curriculum instead of substituting the in-person experience with the virtual session. We appreciate that the home setting offers more expansive opportunities for student growth, including but not limited to IPE alone. There is also the added benefit that students and preceptors alike prefer the in-person method. Going forward, we will incorporate elements of the virtual experience that seem particularly valuable into the in-person home visits (case study, more time to work together independently as a group with minimal preceptor involvement, and reflection on team performance). Our hope is that a blended experience will yield even better learning outcomes and represent the best of both worlds.

## Conclusions

The ultimate aim of our research was to determine if a virtual IPE experience could serve as an alternative pedagogy for students to master IPE objectives required by our curiculum committees for graduation. We demonstrated that even though in-person clinical visits were preferred, the virtual experience was equivalent, if not even more effective, toward meeting IPE competencies.

### Practice points

Medical and pharmacy students preferred in-person clinical learning for this IPE activity.

Virtual learning employing a workshop can provide an alternative teaching strategy to meet IPE learning objectives.

Meeting the IPE learning objectives in the virtual environment may exceed the learning obtained from the clinical environment.

Although students prefer clinical in-person learning, a virtual workshop may offer a viable alternative pedagogy during times of disruption to curriculum delivery.

## Data Availability

Data Archiving and Networked Services: NO PLACE LIKE HOME PROGRAM EVALUATION DATA, https://doi.org/10.17026/dans-xvp-33xz ( Zhou, 2023) This project contains 1 file of underlying data: StudentSurveyDataSchools combined final Data are available under the terms of the Creative Commons Zero "No rights reserved" data waiver (CC0 1.0 Public domain dedication).
