# Supplementary material for: A comparison of medical and pharmacy student perspectives of a clinical interprofessional home-visit versus a virtual interprofessional workshop
Source: MedEdPublish (2016). 2023 May 12;13:27. [Version 1] doi: 10.12688/mep.19510.1 (PMC10331847; doi:10.12688/mep.19510.1)
Supplement: Peer review history

# Peer review: Reviewer response for version 1

**DOI:** 10.21956/mep.20903.r33340

**Published:** 2023-06-27

**Authors:** Heather Hageman, Cynthia Arackal

**Affiliations:** 1 Center for Interprofessional Practice and Education, Washington University Medical Center, St Louis, MS, USA; 2 St. Louis College of Pharmacy, St Louis, Missouri, USA

This research article compares delivery formats by evaluating survey responses of two professions in order to distinguish if one delivery format is superior to the other. Delivery format superiority is determined by specific learning objectives derived from four core domains of the IPE curricula. This research is valuable and relevant to the advancement of student interprofessional learning within the medical field; however, there are a couple of constructive points that are highlighted below.

 The research article’s limitations have only partly been acknowledged. The article addresses challenges with generalizability due to location and a student demographic; however, it does not address limitations with the data collecting method used. Surveys are a subjective form of data that can be easily skewed. Survey participants may differ in their interpretation of the questions, may select answers without reading the questions at all, or may provide responses influenced by (other forms of) fatigue. Although surveys can provide useful data, it is important to be open about the limitations of the data collecting method before drawing conclusions based on the results. Because the data collection method leads to subjective data, it is important to consistently state that any improvements found were perceived. The article insufficiently addresses that the students' improvements were perceived in the section labelled “Which format was preferred, in-person or virtual”. Explicitly stating it consistently throughout the article allows the conclusions to be less misleading.

 Another recommendation is to change the verbiage of the umbrella term used to describe pharmacy students and medical students alike. In the article, “discipline” is the overarching term used to describe the two different types of students involved in the study. This is a common use of the term “discipline”, but relatively imprecise. The term profession would be a more accurate representation of the two different students. An example of two different disciplines would be an internal medicine physician and a pediatric physician, but an example of two different professions would be a physician and a pharmacist.

 This research is valuable within interprofessional practice and education for pharmacy and medical students alike. However, within this specific research article, there remains the aforementioned limitations. The conclusions drawn are solely based on student perceptions of their improved skills. Also, the use of the term discipline in this context should be corrected. This research article has some validity, and it will be exciting to see what other research follows to support these claims.

Have any limitations of the research been acknowledged?

Partly

Is the work clearly and accurately presented and does it cite the current literature?

Yes

If applicable, is the statistical analysis and its interpretation appropriate?

I cannot comment. A qualified statistician is required.

Are all the source data underlying the results available to ensure full reproducibility?

Yes

Is the study design appropriate and is the work technically sound?

Yes

Are the conclusions drawn adequately supported by the results?

Partly

Are sufficient details of methods and analysis provided to allow replication by others?

Yes

Reviewer Expertise:

NA

We confirm that we have read this submission and believe that we have an appropriate level of expertise to confirm that it is of an acceptable scientific standard, however we have significant reservations, as outlined above.

# Peer review: Reviewer response for version 1

**DOI:** 10.21956/mep.20903.r33338

**Published:** 2023-05-25

**Authors:** Maria Wamsley

**Affiliations:** 1 Department of Medicine, and Director, Program for Interprofessional Practice and Education, University of California San Francisco, San Francisco, California, USA

This article looks at student perceptions of an IPE curriculum in 2 different formats - the first was an 8 hour home visit curriculum and the second was a virtual 2 hour case-based small group session that was implemented in the context of the COVID 19 pandemic. Participants in the experience were clinical level pharmacy and medical students.

- I am assuming that the learning objectives for both curricular experiences were the same, though this wasn't explicitly stated in the paper.
- For the evaluation instrument, the authors selected the ICCAS survey which asks students about their skills in 10 different interprofessional competency domains before and after participation in an experience. Additionally there were open ended questions.
- While the authors are to be commended for using an instrument that was developed previously, it is important to acknowledge that this instrument through its measurement of self-assessed IP competencies is a limitation of this study. We know that self-assessment is not always an accurate measure of skill level. The authors at one point state, " *The results provide evidence that the NPLH program enhances students’ interprofessional skills, regardless of discipline and/or delivery format.*" However, I think this would be better phrased as "self-reported IP skills".
- In the methods section the authors talk about a qualitative analysis, but it appears that they have actually done a comment count of the relative frequencies that specific themes came up. Their "qualitative analysis" mentions 3 domains with themes in each of the domains. It would be helpful to better understand why these 3 domains were chosen. Was there a specific theoretical framework the authors had in mind?
- For Table 2 it would be helpful to include the Likert scale and to include the stem for the question for all the items.
- In the section on "Which format was preferred, in-person or virtual" - I find this a bit problematic. It doesn't seem like students were directly asked this question and each group only participated in either the virtual or in person curriculum so hard for them to compare what they didn't experience. I am not sure that it is fair to say that because students who participated in the home visit didn't mention in the comments that they wished it had been virtual is enough to conclude that students preferred the in person version.
- Also in that same paragraph the authors remark " *We observed 4 common differences....*" I am not sure where the points that follow come from - were these from the open ended questions or some other source of data that led to these observations. It would just be important to be clear on this point.
- In the section on "Why do pharmacy students seem to benefit more from the NPLH program?" I found the first sentence a bit odd: " *It appears the pharmacy students responded more thoughtfully to the survey in their text comments and provided more insightful thoughts/opinions.*" I am not sure that the more thoughtful comments means that they benefitted more from the experience. Maybe medical students have evaluation fatigue or pharmacy students have different program expectations around completing evaluations. It felt like a bit of a leap.

Have any limitations of the research been acknowledged?

Partly

Is the work clearly and accurately presented and does it cite the current literature?

Yes

If applicable, is the statistical analysis and its interpretation appropriate?

I cannot comment. A qualified statistician is required.

Are all the source data underlying the results available to ensure full reproducibility?

Yes

Is the study design appropriate and is the work technically sound?

Yes

Are the conclusions drawn adequately supported by the results?

Partly

Are sufficient details of methods and analysis provided to allow replication by others?

Yes

Reviewer Expertise:

Interprofessional education

I confirm that I have read this submission and believe that I have an appropriate level of expertise to confirm that it is of an acceptable scientific standard, however I have significant reservations, as outlined above.

# Peer review: Reviewer response for version 1

**DOI:** 10.21956/mep.20903.r33336

**Published:** 2023-05-25

**Authors:** John C. Luk

**Affiliations:** 1 Dell Medical School, The University of Texas at Austin, Austin, Texas, USA

I am grateful for the invitation and opportunity to review this manuscript. I echo the challenges that colleagues have faced to deliver high quality, interprofessional experiential learning in the face of a pandemic.

 I appreciate that the authors sought to rethink a successful interprofessional experience into a virtual one. Along the way, they have gained insights and learnings elaborated in the manuscript that importantly highlight the dynamics of different learner populations, virtual learning, and curricula. I think that in particular the described learning activity brings learners together to learn from and with each other. I wonder if they expressed learners about each other and their respective professions.

 I think that the learnings are more controlled in a virtual and case-based setting which yielded the desirable result of more interprofessional learnings compared to a live, in-person home visit. I do echo the authors' elaboration of the richness of such an in-person experience and the ad hoc interactions that could result going to, during, and after the encounters. I wonder what the role and/or expectations were of the pharmacy students in the activity as they likely had less clinical experience compared to their medical student peers. Nonetheless, I think that the virtual learning opportunity highlighted or more likely flattened that distinction by enabled them to focus more on their interactions and teamwork over the socio-environmental facets of an in-person encounter.

 I also noted that pharmacy students were supervised by non-pharmacy faculty members (though this was not apparent in the manuscript) and wondered how that aligned with the pharmacy curriculum and expectations. Additionally, I wondered how students were prepared to engage in this experience (virtual and/or in-person). I appreciate that situating the experience in the senior year of medical school might not be ideal and would welcome the authors' continued innovation to right-fit the opportunity in the curriculum.

 I look forward to learning from their continued efforts. Thank you.

Have any limitations of the research been acknowledged?

Yes

Is the work clearly and accurately presented and does it cite the current literature?

Yes

If applicable, is the statistical analysis and its interpretation appropriate?

Yes

Are all the source data underlying the results available to ensure full reproducibility?

Yes

Is the study design appropriate and is the work technically sound?

Yes

Are the conclusions drawn adequately supported by the results?

Yes

Are sufficient details of methods and analysis provided to allow replication by others?

Yes

Reviewer Expertise:

Interprofessional education; professional identity formation; wellness/human flourishing

I confirm that I have read this submission and believe that I have an appropriate level of expertise to confirm that it is of an acceptable scientific standard.
